# Structural Characterization of Neutral Glycosphingolipids from 3T3-L1 Adipocytes

**DOI:** 10.1007/s11745-015-4035-7

**Published:** 2015-05-28

**Authors:** Hisao Kojima, Yusuke Suzuki, Masahiro Ito, Kazuya Kabayama

**Affiliations:** College of Life Science, Ritsumeikan University, Shiga, Japan; College of Science and Technology, Nihon University, Tokyo, Japan; Graduate School of Science, Osaka University, 1-1 Machikaneyama, Toyonaka, Osaka 560-0043 Japan

**Keywords:** Glycosphingolipids, 1,2-Dichloroethane wash, Adipocytes, Triacylglycerols, Liquid chromatography–tandem mass spectrometry

## Abstract

In recent years, obesity has been considered a pathological stage of early lifestyle-related diseases, and adipose tissue and adipocyte research has been active. Glycosphingolipids are involved in the pathogenesis of type 2 diabetes induced by insulin resistance, but the details of the glycosphingolipid molecular species composition of adipocytes have yet to be elucidated. We used 3T3-L1 adipocytes and the 1,2-dichloroethane-wash method to remove triacylglycerols, which are abundant in adipocytes, and analyzed the structures of glycosphingolipids, particularly neutral glycosphingolipids, using liquid chromatography–mass spectrometry.

**Electronic supplementary material:**

The online version of this article (doi:10.1007/s11745-015-4035-7) contains supplementary material, which is available to authorized users.

## Introduction

3T3-L1 cells, a cloned subline of mouse 3T3 fibroblasts, are preadipocytes capable of differentiation into adipocytes [1]. When cocultured with insulin, IBMX, and dexamethasone, they differentiate into adipocytes containing lipid droplets [2]. Adipose tissue is an endocrine organ secreting various adipocytokines, including TNFα [3]. Obesity induces abnormal production of adipocytokines, probably contributing to development of various lifestyle-related diseases, including type 2 diabetes [4]. Hence, studies on adipose tissues and *in vitro* adipocytes for elucidating lifestyle-related disease pathogenesis are necessary.

Glycosphingolipids (GSL), expressed on cell surface membranes, are glycolipid complexes consisting of ceramides linked to a sugar chain. GSL exist in 2 forms: neutral and acidic GSL. Gangliosides are representative acidic GSL, which contains sialic acid residues. Gangliosides are highly expressed in various tissues and are involved in cell differentiation regulation including that of nerve cells [5]. In contrast, neutral GSL are expressed as minor fractions, serving as precursors for gangliosides and glycosyl donors of cholesteryl glucosides and also involved in cytoprotection, myelinogenesis, cell adhesion, and recognition of the integumentary, cardiovascular, nervous, and immune systems [6–8]. GSL may play a critical role in multicellular organisms because the absence of vast majority of GSL derived from glucosylceramide causes embryonic lethality in mice [9].

The analysis of GSL in adipocytes and adipose tissues is difficult because triacylglycerols (TAG) are present in larger amounts compared with GSL, with a few cases reported thus far. These include a lipid structural analysis of the GSL of the pig hind section (loin) using classical methods after degreasing the abundant TAG with acetone [10], two-dimensional thin-layer chromatography (TLC) of fat tissues from various animals [11], TLC of the visceral GSL of the pig peritoneum (omental lipids) [12], TLC of GSL in caveolae and surrounding plasma membrane of primary rat adipocytes [13], liquid chromatography–tandem mass spectrometry (LC/MS/MS) of the glucosylceramide levels in mouse epididymal fat [14], and LC/MS/MS of ganglioside levels in mouse epididymal and ovarian fat [15]. No detailed analysis of molecular species was performed in these studies. We elucidated the role of GSL in type 2 diabetes pathogenesis using TNFα-induced insulin resistant 3T3-L1 adipocytes [16]. During the process, we developed a novel 1,2-dichloroethane (DCE)-wash method, which almost completely removes detergents from detergent-resistant membrane microdomains [17]. In this study, we used LC/MS/MS to characterize the neutral GSL molecular species composition of 3T3-L1 adipocytes using the DCE-wash method.

## Materials and Methods

### Materials

Neutral GSL and monosialoganglioside mixtures were purchased from Matreya LLC (PA, USA). Chloroform, methanol, and DCE of special reagent grade were purchased from Wako Pure Chemical Industries, Ltd (Tokyo, Japan) for lipid extraction and purification. For LC/MS/MS, ammonium hydrogen carbonate (proteomics grade, Wako Pure Chemical Industries, Ltd, Japan), and LC–MS-grade methanol, 2-propanol, and water (Sigma-Aldrich, USA) were used.

### Cell Culture

3T3-L1 fibroblasts were differentiated into adipocytes as described previously [16]. Briefly, we cultured the fibroblasts in D-MEM containing 10 % calf serum until subconfluent, replaced the medium with fresh D-MEM containing 10 % FBS, and cultured them further for 3 days. Fibroblasts were differentiated with supplemental IBMX, dexamethasone, troglitazone, and insulin.

### Lipid Extraction and DCE Washing

3T3-L1 adipocytes were washed with phosphate-buffered saline (PBS) and harvested into screw-capped centrifugal glass tubes. Total lipids were extracted using chloroform/methanol/PBS, (1:2:0.8, v/v) and chloroform/methanol (C/M) 1:2, C/M 1:1, and C/M 2:1. We dissolved the delipidated residue in 0.5 N NaOH, diluted it five times with water, and conducted protein quantification by the bicinchoninic acid method. DCE washing was performed as described previously [16]. Briefly, we dried total lipids under N_2_ stream to maximally cover the inner surface of the glass tube by dried lipid. Highly hydrophobic materials among the dried lipids were washed off 3 times with 2 ml of ice-cold DCE by pipetting. In DCE-wash, the resulting residual fraction was subjected to mild alkali hydrolysis, in which phospholipids were broken down and removed, followed by desalination with Sep-pakC18 and TLC.

### TLC

Samples dissolved in C/M 1:1 were developed on a silica gel HPTLC plate (Merck, Germany) in C/M/0.2 % CaCl_2_ at 60:40:9 (v/v) and visualized by spraying primuline reagent for general lipid and orcinol–H_2_SO_4_ reagent for GSL, or primuline reagent and cupric sulfate reagent.

### LC/MS/MS

Semipurified samples dissolved in LC–MS-grade methanol were analyzed on LCMS-IT-TOF quadrupole ion trap time-of-flight mass spectrometer (Shimadzu, Japan) according to Kuwahata *et al*. [18].

## Results and Discussion

**Fig. 1:**
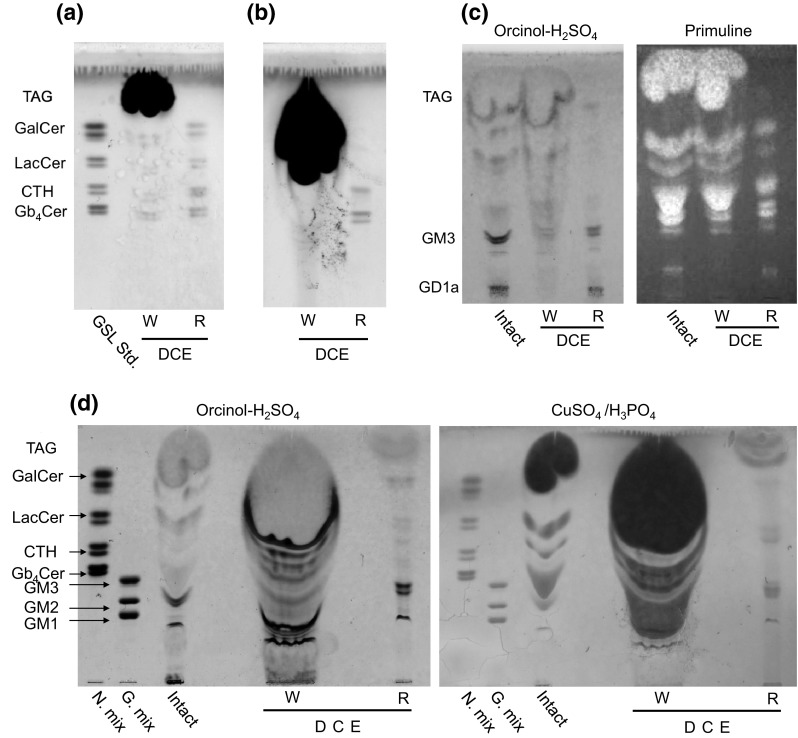
Thin layer chromatogram showing removal of TAG by DCE-wash. **a** Authentic GSL mixture with mouse epididymal fat was dried using a nitrogen stream, washed by pipetting with DCE, and separated into DCE-soluble (*W*) and residual (*R*) fractions. TAG were completely fractioned into the *W* fraction. TLC plate was developed by *C*/*M*/*W*, 60:40:10 (v/v/v) and visualized with cupric phosphate reagent. **b** Large amounts of isolated adipose tissue (mice epididymal fat) were washed with DCE as above. DCE washing also completely fractioned large amounts of TAG into the *W* fraction. GSL-like bands derived from epididymal fat appeared in the *R* fraction. **c** TAG-removal by DCE-wash of TNFα-treated 3T3-L1 adipocytes. Total lipid extract from adipocytes was washed with DCE, developed with C/M/0.2 % CaCl_2_, 60:40:9 (v/v/v) and visualized by orcinol–H_2_SO_4_ and primuline reagents. **d** Large amounts of TNFα-treated 3T3-L1 adipocyte were washed with DCE as above. Neutral GSL, including CMH and CDH, derived from TNFα-treated 3T3-L1 adipocyte appeared even by orcinol-H_2_SO_4_ staining

**Fig. 2:**
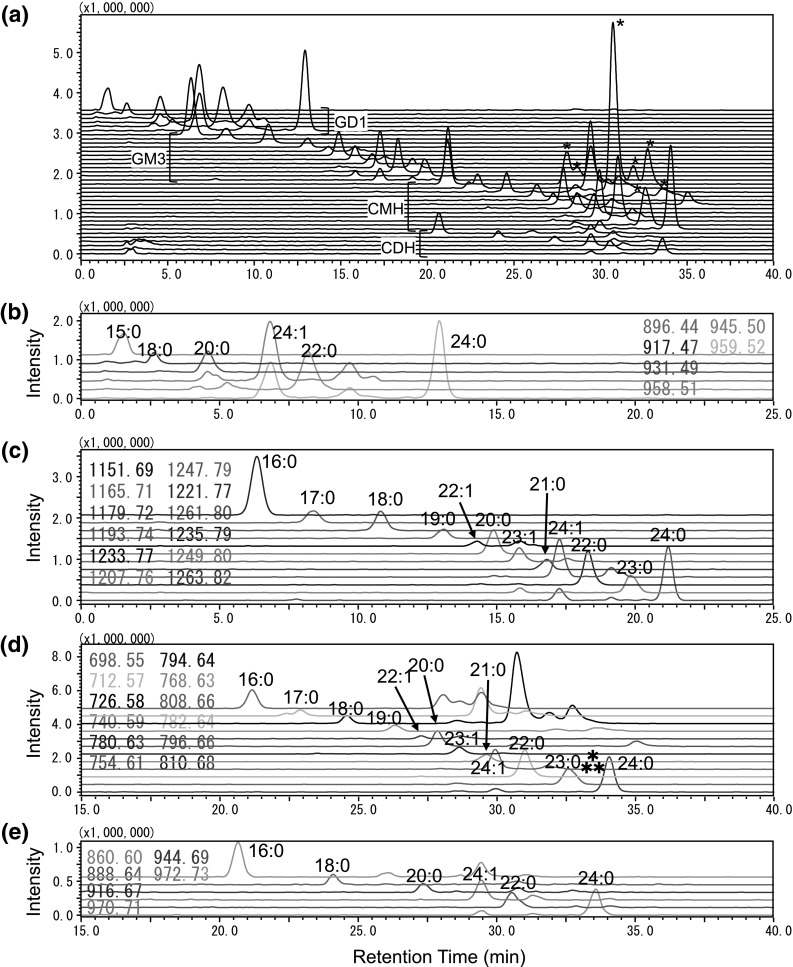
LC–MS chromatogram of major GSL from adipocytes recorded in negative ion mode. Mass chromatogram of whole GSL: (**a**), GD1 (**b**), GM3 (**c**), CMH (**d**), and CDH (**e**). *Asterisks* indicate non-GSL peaks in (**a**). *Asterism* in (**d**) indicates that this peak was composed of two species of CMH dc41:1, namely CMH d18:1-C23:0 and CMH d17:1-C24:0

Under the assumption that TAG were the dominant component of total lipids, we obtained mouse epididymal fat and applied the DCE-wash method to test the removal of TAG by our DCE-wash method. We observed that TAG were removed completely by this method (Fig. 1a, b). Next, we applied DCE-wash to 3T3-L1 adipocytes (Fig. 1c). TAG were efficiently removed and GSL-like bands appeared. However, phospholipid-like bands were also detected in abundance just above GM3. Alkaline hydrolysis was necessary to remove the phospholipids. After removing phospholipids via alkaline hydrolysis, groups of bands probably corresponding to CMH and CDH appeared in the residual fraction (Fig. 1d). However, large amounts of GSL were washed off in the DCE-soluble fraction, which may have been caused by large amounts of adipocyte-derived total lipids used for DCE-wash, containing high concentrations of TAG. A sufficient surface area inside the test tube for dried and solidified GSL adhesion is important in DCE-wash. GSL that did not adhere to the test tube wall appear to have been washed away with TAG. This can be prevented by increasing the surface area for solidification, an important step in DCE-wash. Although most GSL were found in the DCE soluble fraction, the 2 GM3 bands were not changed by DCE washing. This indicates that there was no selective loss of molecular species by DCE-wash. We performed molecular species analysis of GSL in the residual fraction using negative ion mode LC/MS/MS (Fig. 2; S1b, S1c, S2; Table 1). Here to define the structures of GSL, we used a device (Shimadzu LCMS-IT-TOF) capable of performing structural analysis by MS^n^. We set the molecular weight of ceramide in MS^3^ and checked the appearances of fatty acid and long-chain base fragments (P, R and V, U, T, S; Figs. S1, S2) to identify molecular species. In adipocytes, the major GSL was the GM3 ganglioside, followed by GD1a, CMH, and CDH (Fig. 2); molecular species with d18:1 long-chain base were the most abundant and saturated C16–C24 fatty acids predominated.

**Table 1:** GSL species identified in adipocytes

FA	CMH		CDH		GM3		GD1	
	Measured (*m/z*) [M-H]^−^	Calculated (*m/z*) [M-H]^−^	Measured (*m/z*) [M-H]^−^	Calculated (*m/z*) [M-H]^−^	Measured (*m/z*) [M-H]^−^	Calculated (*m/z*) [M-H]^−^	Measured (*m/z*) [M-2H]^2−^	Calculated (*m/z*) [M-2H]^2−^
15:0							896.45	896.45
16:0	698.55	698.56	860.62	860.61	1151.70	1151.71		
17:0	712.57	712.57			1165.71	1165.72		
18:0	726.58	726.59	888.67	888.64	1179.73	1179.74	917.47	917.48
19:0	740.60	740.60			1193.74	1193.75		
20:0	754.61	754.62	916.67	916.67	1207.76	1207.77	931.49	931.49
21:0	768.63	768.64			1221.78	1221.78		
22:1	780.62	780.64			1233.76	1233.78		
22:0	782.65	782.65	944.70	944.70	1235.79	1235.80	945.50	945.51
23:1	794.64	794.65			1247.80	1247.80		
23:0	796.66	796.67			1249.81	1249.81		
24:1	808.66	808.67	970.72	970.72	1261.81	1261.81	958.51	958.52
24:0	810.68	810.68	972.73	972.74	1263.82	1263.83	959.52	959.53

GSL were composed of d18:1 long-chain base and fatty acid moiety indicated in the table below. Molecular species with even-number were dominant, ranging from 16–24. Fatty acid species are indicated by a shorthand notation; the number before the colon indicates the total number of carbon and that after the colon indicates the number of double bonds. ex CMH 16:0 indicate ceramide monosaccharide species with palmitic acid

Determination of the structures of GSL using column chromatography can be difficult because TAG increase the viscosity of the total lipid fraction. Hence, the structural analysis of lipid molecular species is challenging, and detailed analysis of adipose tissue and adipocyte has not been performed thus far. However, owing to their association with the development of lifestyle-related diseases, including insulin resistance, adipocytes have received increasing attention. Studies of GSL in adipocytes focused mainly on quantitative changes in gangliosides, which can be analyzed by column fractionation because of the negative charges of sialic acids unlike TAG. However, the importance of ceramide composition has been indicated in recent years, and studies on qualitative changes in GSL are also desirable. Here we performed a detailed molecular species composition analysis of GSL in adipocytes and presented a method of analysis, which warrants future research on qualitative changes in GSL.

## Electronic Supplementary Material

Supplemental Fig. 1 Fragmentation pattern and mass spectrometry.**a** Ceramide fragmentation diagram. Annotations by Lee *et al*. with slight modification. **b** Mass spectra of CMH 24:0 by LC/MS/MS. CMH was detected at *m*/*z* 810.68 and fragmented by MS/MS. A resulting MS^2^ showed a ceramide moiety peak at *m*/*z* 648.62, further fragmented by MS^3^. A resulting MS^3^ showed a LCB-derived ion (P and R) and FA-derived ions (V, U, T, and S). (c) Mass spectra of CDH 24:0 by LC/MS. CDH was detected at *m*/*z* 972.73 and fragmented by MS^2^. MS^2^ spectrum showed ceramide moiety ion at *m*/*z* 648.62, further fragmented by MS^3^. The MS^3^ spectrum showed FA-derived ions (U, T, and S). Supplemental Fig. 2 Fragmentation pattern of molecular species composed of odd-number fatty acids. (a) Mass spectra of GD1 15:0 by LC/MS/MS. GD1 was detected at *m*/*z* 896.45 and fragmented by MS/MS. A resulting MS^2^ showed a desialylated peak at *m*/*z* 1502.8 and a fragment peak corresponding to ceramide disaccharide at *m/z* 846.6. (b) Mass spectra of GM3 23:0 by LC/MS/MS. GM3 was detected at *m*/*z* 1249.80 and fragmented by MS/MS. A resulting MS^2^ showed fragment peaks corresponding to ceramide disaccharide and ceramide monosaccharide at *m*/*z* 958.7and 796.7, respectively, and a ceramide moiety peak at *m*/*z* 634.6. (c) Mass spectra of CMH 23:0 by LC/MS/MS. CMH was detected at *m*/*z* 796.66 and fragmented by MS/MS. A resulting MS^2^ showed a ceramide moiety peak at *m*/*z* 634.6, further fragmented by MS^3^. A resulting MS^3^ showed C23 fatty acid-derived ions (V, U, T, and S) (PDF 139 kb)

## Abbreviations

TAG: Triacylglycerol(s)
DCE: 1,2-Dichloroethane
LC/MS/MS: Liquid chromatography/tandem mass spectrometry
TLC: Thin-layer chromatography
GSL: Glycosphingolipid(s)
CMH: Ceramide monohexoside
CDH: Ceramide dihexoside

## References

[1] Green H, Kehinde O (1974). Sublines of mouse 3T3 cells that accumulate lipid. Cell.

[2] Rubin CS, Hirsch A, Fung C, Rosen OM (1978). Development of hormone receptors and hormonal responsiveness *in vitro*. Insulin receptors and insulin sensitivity in the preadipocyte and adipocyte forms of 3T3-L1 cells. J Biol Chem.

[3] Galic S, Oakhill JS, Steinberg GR (2010). Adipose tissue as an endocrine organ. Mol Cell Endocrinol.

[4] Cawthorn WP, Sethi JK (2008). TNF-α and adipocyte biology. FEBS Lett.

[5] Kolter T (2012). Ganglioside biochemistry. ISRN Biochem.

[6] Boggs JM, Gao W, Hirahara Y (2008). Myelin glycosphingolipids, galactosylceramide and sulfatide, participate in carbohydrate–carbohydrate interactions between apposed membranes and may form glycosynapses between oligodendrocyte and/or myelin membranes. Biochim Biophys Acta.

[7] Hakomori S (2008). Structure and function of glycosphingolipids and sphingolipids: recollections and future trends. Biochim Biophys Acta.

[8] Messner MC, Cabot MC (2010) Glucosylceramide in humans. In: Chalfant C, Del Poeta M (ed) Sphingolipids as signaling and regulatory Molecules. Advances in experimental medicine and biology. Springer, New York 688:156–164 10.1007/978-1-4419-6741-1_11 20919653

[9] Yamashita T, Wada R, Proia RL (2002). Early developmental expression of the gene encoding glucosylceramide synthase, the enzyme controlling the first committed step of glycosphingolipid synthesis. Biochim Biophys Acta.

[10] Ohashi M, Yamakawa T (1977). Isolation and characterization of glycosphingolipids in pig adipose tissue. J Biochem.

[11] Ohashi M (1979). A comparison of the ganglioside distribution of fat tissues in various animals by two-dimensional thin layer chromatography. Lipids.

[12] McCluer RH, Evans JE, Kamarei AR, Williams MA (1989). Characterization of porcine omental lipids. Lipids.

[13] Örtegren U, Karlsson M, Blazic N, Blomqvist M, Nystrom FH, Gustavsson J, Fredman P, Strålfors P (2004). Lipids and glycosphingolipids in caveolae and surrounding plasma membrane of primary rat adipocytes. Eur J Biochem.

[14] Wu D, Ren Z, Pae M, Guo W, Cui X, Merrill AH, Meydani SN (2007). Aging up-regulates expression of inflammatory mediators in mouse adipose tissue. J Immunol.

[15] Tanabe A, Matsuda M, Fukuhara A, Miyata Y, Komuro R, Shimomura I, Tojo H (2009). Obesity causes a shift in metabolic flow of gangliosides in adipose tissues. Biochem Biophys Res Commun.

[16] Kabayama K, Sato T, Saito K, Loberto N, Prinetti A, Sonnino S, Kinjo M, Igarashi Y, Inokuchi J (2007). Dissociation of the insulin receptor and caveolin-1 complex by ganglioside GM3 in the state of insulin resistance. Proc Natl Acad Sci U S A.

[17] Suzuki Y, Kabayama K (2012). Convenient and rapid removal of detergent from glycolipids in detergent-resistant membrane microdomains. J Lipid Res.

[18] Kuwahata H, Yamaguchi T, Kojima H, Kabayama K (2014). Atmospheric-pressure plasma jet irradiation onto main components of the cell wall and membrane of *Escherichia coli*. e-J Surf Sci Nanotech.

