# Supplementary figures and images for: Structural Characterization of Neutral Glycosphingolipids from 3T3-L1 Adipocytes
Source: Lipids. 2015 May 28;50(9):913–7. doi: 10.1007/s11745-015-4035-7 (PMC4541715; doi:10.1007/s11745-015-4035-7)

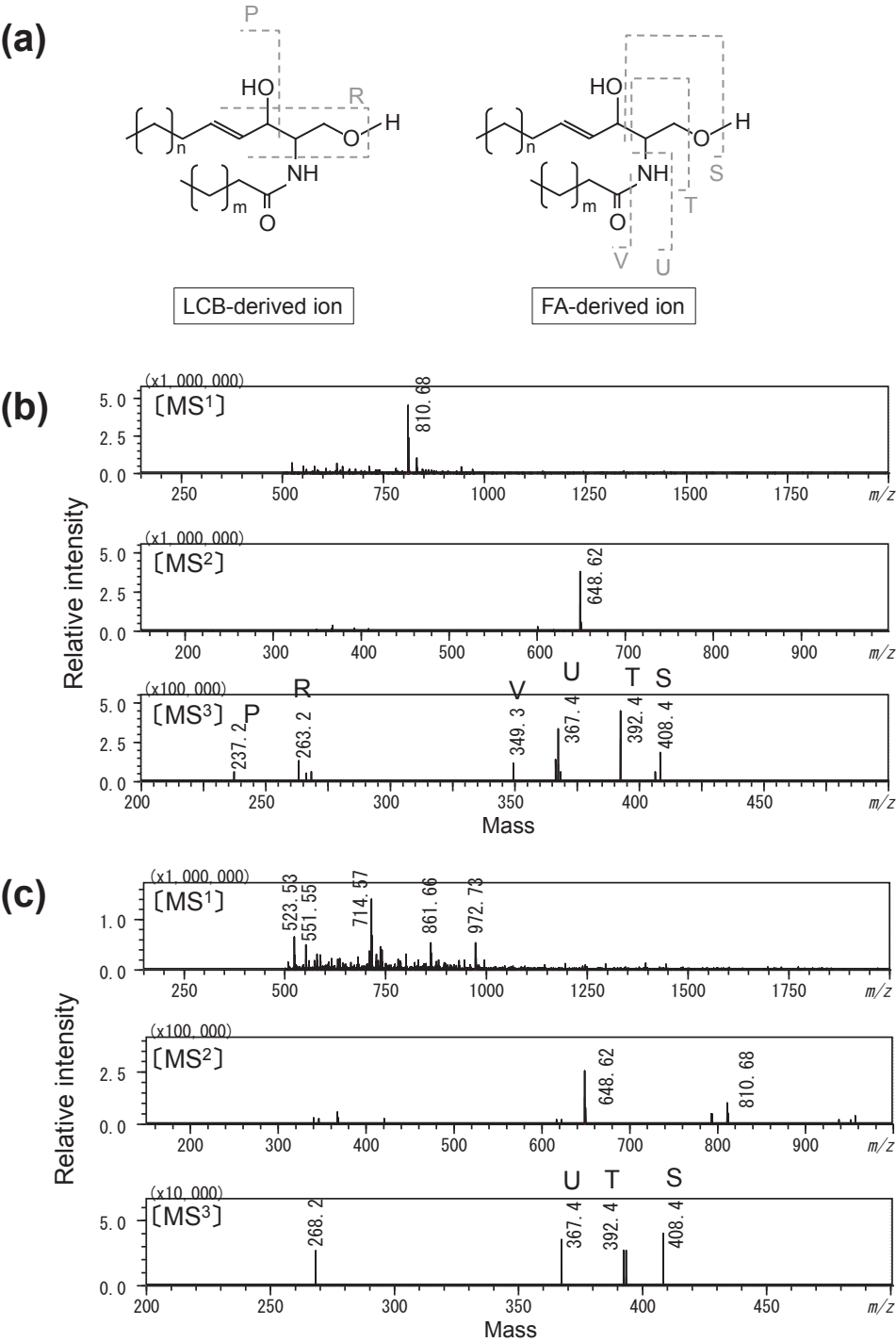

Suppl. Fig.1

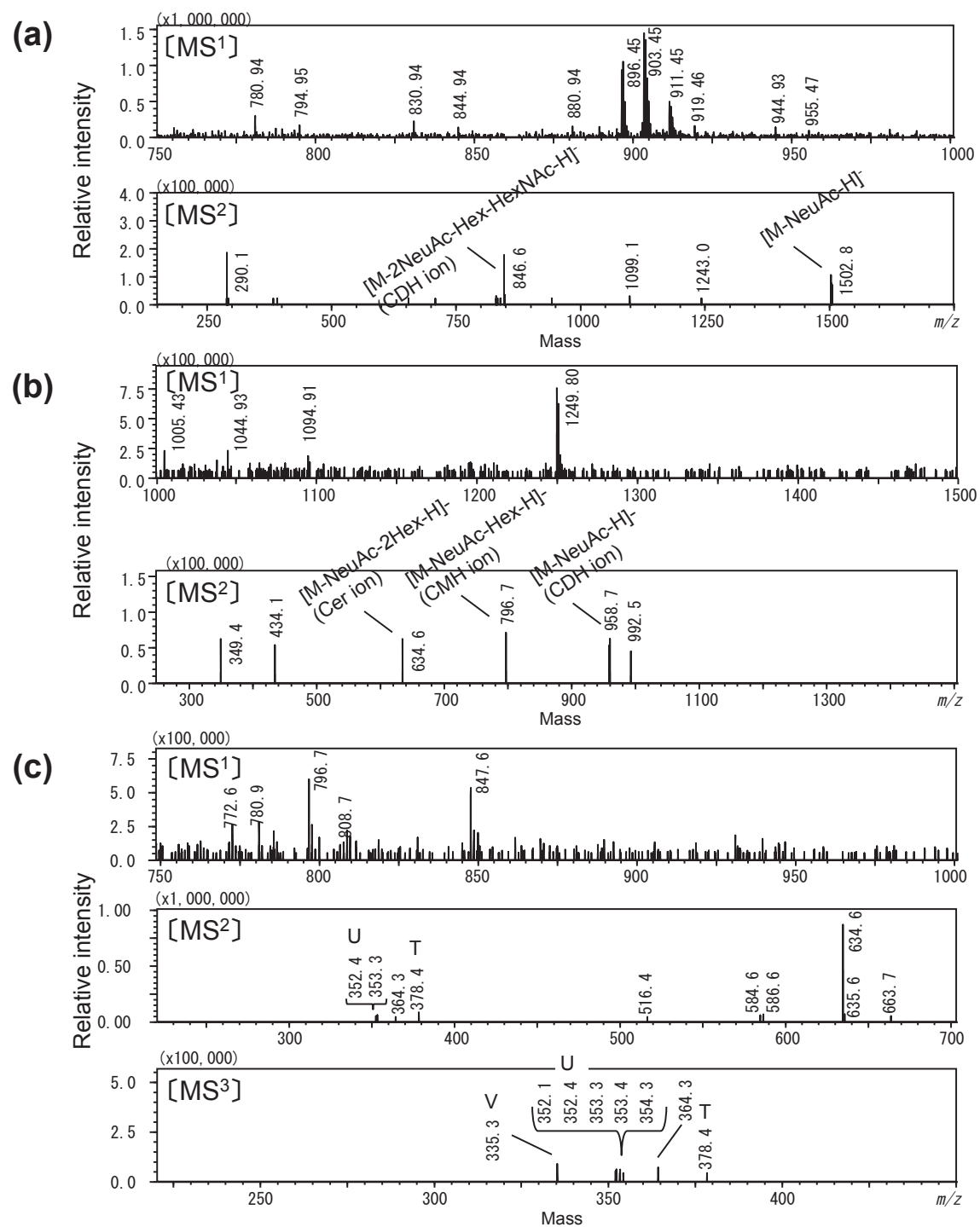

Suppl. Fig.2

Supplement: Supplementary file 1 — Supplemental Fig. 1 Fragmentation pattern and mass spectrometry.a Ceramide fragmentation diagram. Annotations by Lee et al. with slight modification. b Mass spectra of CMH 24:0 by LC/MS/MS. CMH was detected at m/z 810.68 and fragmented by MS/MS. A resulting MS2 showed a ceramide moiety peak at m/z 648.62, further fragmented by MS3. A resulting MS3 showed a LCB-derived ion (P and R) and FA-derived ions (V, U, T, and S). (c) Mass spectra of CDH 24:0 by LC/MS. CDH was detected at m/z 972.73 and fragmented by MS2. MS2 spectrum showed ceramide moiety ion at m/z 648.62, further fragmented by MS3. The MS3 spectrum showed FA-derived ions (U, T, and S). Supplemental Fig. 2 Fragmentation pattern of molecular species composed of odd-number fatty acids. (a) Mass spectra of GD1 15:0 by LC/MS/MS. GD1 was detected at m/z 896.45 and fragmented by MS/MS. A resulting MS2 showed a desialylated peak at m/z 1502.8 and a fragment peak corresponding to ceramide disaccharide at m/z 846.6. (b) Mass spectra of GM3 23:0 by LC/MS/MS. GM3 was detected at m/z 1249.80 and fragmented by MS/MS. A resulting MS2 showed fragment peaks corresponding to ceramide disaccharide and ceramide monosaccharide at m/z 958.7and 796.7, respectively, and a ceramide moiety peak at m/z 634.6. (c) Mass spectra of CMH 23:0 by LC/MS/MS. CMH was detected at m/z 796.66 and fragmented by MS/MS. A resulting MS2 showed a ceramide moiety peak at m/z 634.6, further fragmented by MS3. A resulting MS3 showed C23 fatty acid-derived ions (V, U, T, and S) (PDF 139 kb) [file 11745_2015_4035_MOESM1_ESM.pdf]
